# Phage based vaccine: A novel strategy in prevention and treatment

**DOI:** 10.1016/j.heliyon.2023.e19925

**Published:** 2023-09-15

**Authors:** Sharareh Mohammad Hasani, Elham Ghafouri, Shirin Kouhpayeh, Forouzan Amerizadeh, Ilnaz Rahimmanesh, Zohre Amirkhani, Hossein Khanahmad

**Affiliations:** a Department of Genetics and Molecular Biology, School of Medicine, Isfahan University of Medical Sciences, Isfahan, Iran; b Erythron Genetics and Pathobiology Laboratory, Department of Immunology, Isfahan, Iran; c Department of Neurology, Mashhad University of Medical Sciences, Mashhad, Iran; d Department of Internal Medicine, Faculty of Medicine, Mashhad University of Medical Sciences, Mashhad, Iran; e Applied Physiology Research Center, Cardiovascular Research Institute, Isfahan University of Medical Sciences, Isfahan, Iran

**Keywords:** Bacteriophage, Vaccine, Phage-based vaccine

## Abstract

The vaccine was first developed in 1796 by a British physician, Edward Jenner, against the smallpox virus. This invention revolutionized medical science and saved lives around the world. The production of effective vaccines requires dominant immune epitopes to elicit a robust immune response. Thus, applying bacteriophages has attracted the attention of many researchers because of their advantages in vaccine design and development. Bacteriophages are not infectious to humans and are unlikely to bind to cellular receptors and activate signaling pathways. Phages could activate both cellular and humoral immunity, which is another goal of an effective vaccine design. Also, phages act as an effective adjuvant, along with the antigens, and induce a robust immune response. Phage-based vaccines can also be administered orally because of their stability in the gastrointestinal tract, in contrast to common vaccination routes, which are intradermal, subcutaneous, or intramuscular. This review presents the current improvements in phage-based vaccines and their applications as preventive or therapeutic vaccines.

## 1 Introduction

The vaccine innovation by Edward Jenner against smallpox [1,2] has revolutionized medicine during the last centuries and saved the lives of humanity and many animals [3,4]. The vaccines are classified into various categories based on their nature, including inactivated, live-attenuated, and recombinant vaccines [3,5]. Although remarkable advances have been made regarding conventional vaccines, some limitations, such as the high cost and low immune responses, have been reported. Therefore, novel strategies, such as phage-based vaccines, have been developed to overcome the limitations of classical vaccines [3,6]. Phage-based vaccines elicit an effective cellular and humoral immune response with no need for adjuvants and no potential risk of infection in human cells or integration into the human genome [[6], [7], [8]]. Bacteriophages are the most abundant living organisms around the world, and they have a high ability to coexist with a multitude of organisms like mammals; thus, oral administration to humans has been approved by the FDA [[9], [10], [11], [12]]. Phages could also play various roles in drug delivery, phage therapy, biosensors, and biotechnology applications such as the identification of ligand binding sites and B-cell epitopes and the production of monoclonal antibodies in large volumes [6,[13], [14], [15], [16], [17], [18], [19]]. In this regard, recombinant bacteriophage technology provides a cost-effective and potent solution to the increasing demand for efficient vaccines against different pathogens. Herein, we first discuss the basics of phage biology and mechanisms of immunity against engineered phages and then review the three categories of phage-based vaccines, including Phage display vaccines, Phage- DNA vaccines, and Hybrid phage-DNA vaccines; which are under clinical trials or commercially available.

## 2 Bacteriophages

Phages, or bacteriophages, are a class of viruses that infect archaea and bacteria, but they are incompetent for infecting eukaryotes [7,9]. The bacteriophages are divided into RNA phages and DNA phages in terms of genetic material, which is covered by a coating protein called a capsid.

## 3 1. RNA phages

RNA phages in two groups Cystoviridae with double-stranded segmented RNA (ds RNAs) and Leviviridae with single-stranded RNA (ss RNAs) are classified. Leviviridae are easy to produce in large amounts, and because they have a plus-sense single-stranded RNA genome, they served as a convenient source of messenger RNA (mRNA) for studies of protein synthesis. MS2 and Qβ are examples of Leviviridae; both use F-pilus to enter *E. coli* cells [20]. φ6 is an example of Cystoviridae, most of which use type IV pili, and some of them use lipopolysaccharides on the bacterial cell surface to enter the bacterial cell [20].

### 3.1 DNA phages

#### 3.1.1 Non-lytic phages

##### 3.1.1.1 Filamentous phages

**Fig. 1:**
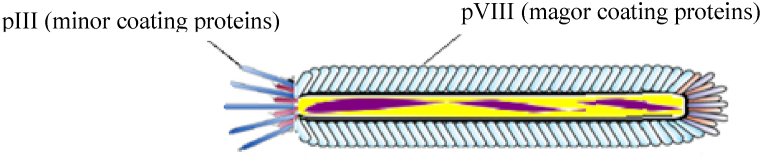
Schematic illustration of Filamentous phages.

Filamentous phages, such as M13, are a group of viruses that are non-lytic and consist of a 6.4 Kb circular ssDNA [10,21]. One of the important advantages of filamentous phages is their workability and inexpensive purification, which can be purified from bacterial culture mediums with high titers [22]. In addition, M13 particles can be used as vaccine carriers because of their immunogenic potential [7,23]. The coating of the particles contains minor coat proteins (pIII, pVI, pVII, and pIX) and major coat proteins (pVIIIs). 2700 copies of pVIII cover the entire particle surface. Among the coat proteins, pIII and pVIII proteins are the most widely used proteins for displaying external peptides, depending on the length and sequence of the peptide in the phage display technique. Immunogenic peptides are fused with PIII or PVIII and displayed on the phage surface (Fig. 1) [9,24]. Although pVIIIs surround the whole viral particle, short peptides of up to 8 amino acids can be fused to them; thus, it is required to maximize the presentation flexibility of the system to be able to display longer peptides on them [23,[25], [26], [27]] or engage pIIIs for larger peptide fragments [[28], [29], [30]]. However, the capability of pVIIIs to display a higher number of immunogenic peptides leads to an enhanced immune response. Another important advantage of filamentous phages is their stability of particles against harsh temperatures and pH variations, so the filamentous phage genome can be applied as a cloning vector for the attachment of external DNA in different sizes [9,29].

#### 3.1.2 Lytic phages

##### 3.1.2.1 T4 phages

**Fig. 2:**
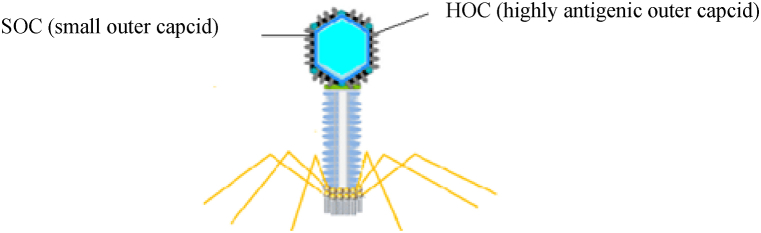
Schematic illustration of T4 phages.

T4 phages are categorized as lytic viruses with a double-stranded DNA (dsDNA) genome about 169 kb in size [31]. Hoc and Soc are high-copy bacteriophage capsid proteins that can display peptides with high copies and larger than the peptides displayed by filamentous phages on their surface (Fig. 2), leading to an effective immune response in mice and humans [11,32,33]. Clinical trial evidence suggests the oral administration of whole wild-type T4 is remarkably safe for humans [11]. Other advantages of T4 phages include their high immunogenicity, non-toxicity of the secreted protein, and ability to express the dual-engineered protein. Therefore, considering the advantages mentioned, T4 application as a phage display vector is more common than filamentous phages [34].

##### 3.1.2.2 T7 phages

**Fig. 3:**
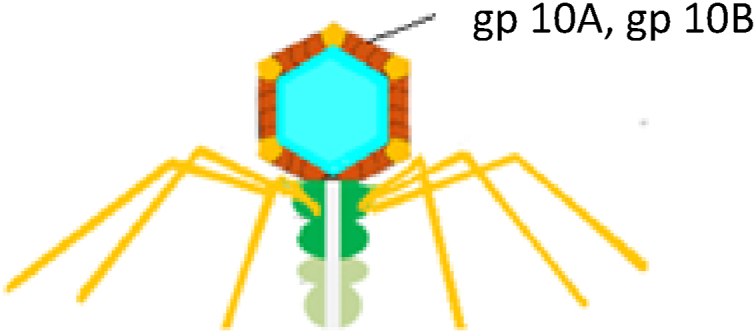
Schematic illustration of T7 phages.

Other lytic viruses include T7 phages. The structure of the T7 phage consists of a linear dsDNA with a size of about 40 kb and six main proteins called capsid proteins (10A and 10B), tail proteins (gp11 and gp12), tail fiber protein (gp17), and connector protein (gp8). Gp 10 A and gp 10 B are often chosen for phage display purposes (Fig. 3) [6,[35], [36], [37]]. The carboxyl-terminus of the 10 B protein of the T7 phage has been engineered, and the recombinant T7 phage was produced to display heterologous proteins and activate the humoral and cellular immune responses [[35], [36], [37], [38]]. The advantages of using recombinant T7 phage include high cloning capacity, high stability to maintain external gene inserts, and a high propagation rate [39].

#### 3.1.3 Temperate phages

##### 3.1.3.1 λ phages

**Fig. 4:**
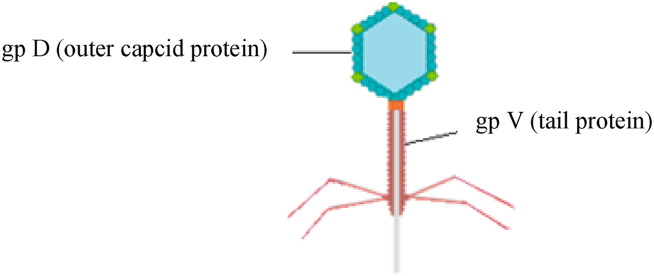
Schematic illustration of lambda phages.

Lambda phages are categorized as temperate phages with a linear dsDNA genome about 48.5 kb in size (Fig. 4) [40]. Lambda phages can display peptides with proper folding, higher density, and a size 2–3 times larger than what filamentous phages display. Antigens on lambda can be exposed by both the head protein pD and the tail protein pV [9]. Although filamentous phages are still one of the most common vectors for phage display, lambda phages could also be a good option for displaying complex antigens [9,41,42]. For instance, beta-galactosidase with about 400 kDa MW was properly displayed on the surface of the lambda phage with no changes in phage structure or survival [26].

## 4 Weaknesses and strengths of phage systems

The M13 phage is the most frequently used phage in the phage display technique because it has non-essential regions that permit exogenous gene insertions and because exogenous peptides express themselves on the phage surface through their natural folding and function [43,44] he most important feature of phage M13 compared to other phages is that it can be purified and used with less effort. In fact, the host bacterium acts as a factory to produce M13 phages [45,46]. However, the M13 phage display is associated with limitations in the construction of the cDNA library, and the formation of a fusion protein comprising phage coat protein and exogenous peptide and the secretion of phage into the bacterial periplasmic space are also potential problems with the M13 phage display. Unlike M13 phages, which contain single-stranded DNA, T7 phages have double-stranded DNA, which makes them less prone to mutation during replication and more stable, and they no longer depend on the protein secretion pathway. The exogenous peptides are usually in the C-terminal region of the gp10B capsid protein, which does not have the problems associated with steric hindrance. Also, T7 phages have high stability and tolerate harsh conditions such as low pH and high temperatures [39]. Lambda phages have both lysogenic and lytic alternatives for their life cycle. The genome of lambda phages consists of linear dsDNA, which becomes circular for replication after infecting *E. coli* bacteria. The limitation of lambda phages as vectors is theire low delivery capacity of about 35–50 kb of DNA [47,48]. Lambda Phage is attached to the surface of *E. coli* cells, which have a place to transfer maltose into the cell. Therefore, to infect bacteria with Lambda phage, the culture medium must contain maltose [49,50] and when the phage DNA enters the bacterial cell, a circular DNA molecule is formed due to the complementary sticky 12 bp sequence at both ends of its genome. 45 min after the infection, the bacterial cells are lysed, the phages are released, and the infection spreads to nearby cells. This cycle is repeated regularly [49,51]. Another thing that should be considered when working with Lambda phage in the laboratory is that, due to the free sticky ends in the Lambda phage genome, under normal conditions these ends can stick together and create a large fragment. To avoid this possibility, it is recommended to heat the Lambda markers before electrophoresis if electrophoresis is necessary during the operation [52]. And for T4 lytic phages, two distinct features have made their use more common than filamentous phages: one is the ability to express dual-engineered proteins, and the other is the high immunogenicity of their capsid [34]. One of the prominent features of the T4 genome is that instead of cytosine (C), 5-hydroxymethylcytosine (HMC) is located. HMC causes the viral enzymes to distinguish between their own and the host's nucleic acids, and the virus genome is protected from the effects of its enzymes. Because the viral enzymes break the host's genome into pieces and take over all the host's facilities. Also, glycosylation happens by adding glucose to HMC, which makes the phage immune to the attack and degradation of bacterial repair systems. It is noteworthy that glycosylation is specific to eukaryotes and is not found in bacteria except for a few exceptions. But this phenomenon happens in T4 phages, which is one of the wonders of creation [[53], [54], [55], [56], [57], [58], [59], [60]]. Additionally, some stability studies showed that T4 remained stable at ambient temperature for at least 10 weeks. As a result, the distribution of T4-based vaccines does not require a cold chain [61,62].

## 5 Phage display technology

**Fig. 5:**
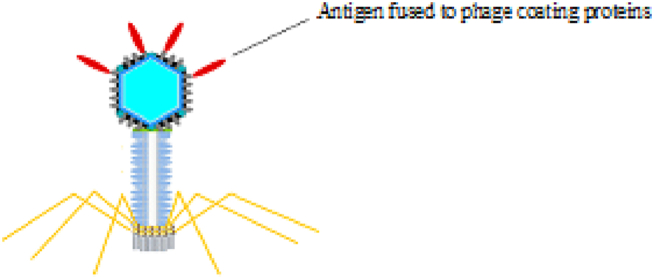
Schematic illustration of phage displayed antigens and phage display vaccines.

In phage display technology, immunogenic peptides are joined to coating proteins and then shown on the surface of the phage (Fig. 5) [7,21,63]. Using bacteriophages as antigen carriers leads to increased antigen half-life in peripheral blood and also enhances the immune response by facilitating the activation of T helper cells [64]. The phage display technique has been used for vaccine research and immunotherapy in the past 10 years, and hopefully it has opened new landscapes for the vaccine industry and the prevention of infectious diseases [21,65]. Phages that have been used to display antigens and develop phage-based vaccines are Lytic, Filamentous, and Lambda phages, which are detailed in Table 1.

**Table 1:** Features, design, application, and main findings after administration of common phage display vaccines.

	VECTOR TYPE	MODEL OF STUDY	PHAGE TYPE	DOSAGE	USED PROTEIN	EFFECTS AFTER ADMINISTRATION/COMMENTS	STRAIN OF E.Coli	REFERENCE
Anti-viral Hepatitis B virus epitope S28–39	pC89	Female BALB/c mice	M13	10 μg i.d i.p	pVIII	MHC I limited response of HBs specific cytotoxic T cells	XL1-blue	[66,66]
Anti-parasite Peptides GK1, KETc12, KETc1, and KETc7 Taenia solium cysticercosis	M13KE	Pig	M13	2 ml,10^12^ phage particle of each sc	pVIII	Reduction of 70% of tongue cysticercoss Reduction of 87% of total cysticerci Reduction of 54% of muscle cysticercosis	TG-1	[15,15]
Anti-viral Epitopes of the glycoprotein G of HSV-2 (gG2)	n.r	BALB/c mice	Fd-tet	n.r	pVIII	Upgrade of protective immunity in a mice model of HSV-2 infection	K91Kan	[25,25]
Anti-cancer EGFR ICR-62 binding peptide mimotope	pAK8-GVO	Female BALB/c mice	M13	10^12^ pfu s.c	pVIII	Decreased tumor growth in ectopic Lewis lung carcinoma in mice Humoral immunity stimulated in mice	TG-1	[18,18]
Anti-viral Ectodomain of influenza A virus channel protein M2 (M2e)	n.r	Female BALB/c mice	T7	10^9^ pfu s.c	10 B capsid protein	M2e-specific serum antibody responses Reduction of viral load due to Cytotoxic T cell response Protection against Influenza A virus	BL21	[35,35]
Hybrid Anti-cancer HLA-DR-restricted Th cell peptide epitope p23 and MAGE-A10254–262 or p23 and MAGE-A3271–279 from the HIV-1-RT	pTfd8p-66 for p23 peptide	Human cell system in vitro Humanized murine model in vivo	fd	140 μg phage particles s.c	pVIII	Inhibited tumor growth due to stimulation of potent and specific cytotoxic T cell response	TG1 rec O	[67,67]
Dual display of swine fever virus (CSFV) major antigenic determinant cluster mE2 and CSFV primary antigen E2	pcDSW	Female BALB/c mice	T4-Zh-	10^10^ pfu s.c	Soc C-terminus fusion and Hoc N-terminus	Increased immune response High antibody production	BL21	[68,68]
*Yersinia pestis* (Plague) capsular F1 and calcium response V antigen	pET-28b	Female BALB/c mice	T4 Hoc-soc-	10 μg phage particles i.m	Soc	Induced Th1 and Th2 response Complete protection against pneumonic plague	BL21	[69,69]
Anti-cancer Melanoma Epitope MAGE-A1161–169	pfd8wf	Mice and cell lines YAC-1 and B16.F10	fd	50 μg phage particles i.p	pVIII	Hypersensitivity type IV Inhibition of tumor growth due to Specific cytotoxic T cell response and enhanced NK activity	TG1	[70,70]
Anti-viral HRSV G glycoprotein-epitope 173–187	Bacteriophagevector Fuse 5	BALB/c mice	fd	1 mg i.p	pIII	Stimulation of strong immune response against RSV infection in mice	K91 Kan	[71,71]
Anti-liver cancer ASPH peptides	pVCDcDL1A	Murine	λ	10^10^ pfu s.c	gpD	Enhanced in CD4^+^ and CD8^+^ response Secretion of Th1 and Th2 Specific cytokine Decreased Hepatocellular carcinoma growth	n.r	[42,42]
Anti-cancer EGFR gene of extracellular domain of chicken xenogeneic EGFR	n.r	Male Kunming mice	T7	2.5 × 10^13^ pfu	10 B	Decreased tumor growth and progression Stimulation of humoral immune response and increase in specific anti-EGFR antibodies	n.r	[72,72]
Contraceptive 3 Gonadotrophin Releasing Hormone (GnRH) fragment 43 kDa	n.r	Male BALB/c mice	T7	10^10^ pfu s.c	10 B	Secretion of Specific anti-GnRH antibody Spermatogenesis suppression	BL21	[73,73]
Anti-cancer Mouse Fms-like tyrosine kinase 4 (Flt4)	T4-Z	Mice	T4	10^11^ pfu s.c	Soc	Prevention of metastasis in Lewis lung carcinom due to stimulation of antitumoral immunity Induction of anti- Flt4 antibody	BL21	[74,74]
Anti-cancer Vascular endothelial growth factor receptor 2 (VEGFR2)	pD-mVEGFR2	Mouse tumor model Male C57BL/6J	T4-SGPDS	5 × 10^11^ pfu s.c	Soc C-terminus fusion	Stimulation of anti-angiogenesis activity Stimulation of anti-tumor immunity Stimulation of CD4^+^ T cell–mediated effector mechanisms	BL21 or HB101	[34]
Anti-bacteria in vitro display Protective antigen (PA) from B. anthracis	pET-15b	Female CBA/J mice	T4 Hoc-soc-	10^10^ pfu i.m	Hoc N-terminus	Neutralization of anthrax lethal toxin due to the production of high levels antibodies	P301 (sup-)	[75,75]

i. p: intraperitoneal; i. d: Intradermal; HRSV: Human Respiratory Syncitial Virus; s.c: subcutaneous; MHC: major histocompatibility complex; pfu: Plaque forming units; ASPH: Aspartateβ-hydroxylase; Herpes Simplex Virus (HSV); n.r.: not reported; EGFR: Epidermal Growth Factor Receptor; i.m: intramuscular; F: Filamentous; HLA-DR: Human Leukocyte Antigen-DR isotype; T4-S-GPDS: T4 bacteriophage nanoparticle surface gene-protein display system; RT: Reverse Transcriptase.

### 5.1 Phage display strategy for vaccine development

As mentioned previously, phage display has been employed as an effective tool for vaccine design and development. This strategy could also be used to produce vectors to identify new antigens with desired biological and physicochemical properties as the first step of vaccine design, in addition to displaying antigens. In the first step of the vaccine design process, after the identification of novel antigens, phage display is employed to produce random peptides through genetic engineering, which leads to the selection of suitable epitopes for stimulation of the immune system.

**In an in vivo phage display system**, the expression of antigens occurs during the phage infection stage [75]. There are problems with the system, such as differences in how proteins are expressed inside cells, incomplete phage assembly, and protein accumulation [75], which leads to improper folding and poor performance as a vaccine [10]. In vitro phage display systems resolved the limitations and problems of the In vivo system, and the protein fusion process was performed with correct folding and more quantity [10].

#### 5.1.1 Production of antigen-displaying vectors

In phage display and phage-based vaccine technology, peptide antigens are displayed in fusion with the phage surface proteins. The resulting recombinant viral particles have specific immunological activity [76]. Anti-cancer phage-based vaccines, antiviral phage-based vaccines, and immunocontraceptive phage-based vaccines are examples of phage-based vaccines. Anticancer vaccines have been effective in cancer immunotherapy in recent years. Some candidate tumor antigens have been assessed as the best immunogenic antigens in anticancer phage-based vaccines, such as Epidermal Growth Factor Receptor (EGFR) [72], Fms-like tyrosine kinase 4 (Flt4), and Melanoma Antigen Gene (MAGE) [77]. The phages used in anti-cancer phage vaccines include filamentous phages in mouse and rabbit tumor models [28] and T4 and M13 phages in mouse tumor models [78]. The anticancer mechanisms used include the death of tumor cells and the reduction of viable tumor cells by inducing the infiltration of neutrophils [79], the reduction of tumor cell proliferation by stimulating the humoral immune system and the production of antibodies [18], and the degradation of tumors by the use of toll-like receptors (TLR) [79]. Among the effective phage-based antiviral vaccines, epitopes of hepatitis B, HIV, herpes simplex virus 1 (HSV-1), herpes simplex virus 2 (HSV-2) [25], human respiratory syncytial virus [71], and circovirus 2 (PCV2) [10] are displayed on the phage surface. Another immunocontraceptive phage-based vaccine that has been used in mouse models for immunocastration is the gonadotropin-releasing hormone (GnRH) antigen on the T7 phage surface [73]. Table 1 shows in detail several phage-based vaccines and the main findings after their administration.

#### 5.1.2 Antigen identification (Phage bio-panning technology)

**Fig. 6:**
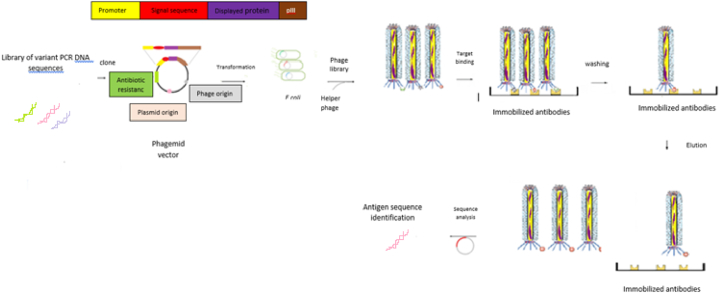
Antigen identification using phage display technology includes the stages of phage library construction, phage selection, and antigen identification.

In this process, random or natural cDNA sequence variants are cloned into the phage genome to express different antigens in fusion with coat proteins on the phage surface and produce libraries of phages carrying different candidate antigens. From the libraries, phages with high affinity for desired targets are selected and their genomes sequenced, and in this way, the encoding sequence for the target antigen is identified [30]. The advantages of this method in vaccine design include rapid identification, low cost, and high efficacy [21]. For the display of antigens in M13, pIII is a better option because the fused antigens (displayed antigens) bind to the desired targets with a higher affinity [30]. The main applications of the bio-panning technique are the identification of linear and continuous epitopes with a length of 4–6 amino acids (Fig. 6) [21,80] and mimotope (a peptide mimicking the structure of an epitope) (Table 2). The application of mimotopes is more advantageous than epitopes because of the facility of synthesis, imitation of non-protein antigens such as carbohydrates and lipids, and high bioactivity [18,28,29,81]. The application of mimotopes was reported to identify amino acid sequences of immunogenic domains in toxins like botulinum neurotoxin A [82]. Table 2 describes some of the epitopes and mimotopes displayed on phages that have developed in vivo effective responses in mouse models.

**Table 2:** Examples of identification of epitopes, antigens, and mimotopes by bio-panning technology, describing the type of vector used (phage or phagemid), the main findings such as screening antibodies, and their therapeutic or prophylactic results.

SOURCE OF ANTIGEN	BACTERIOPHAGE VECTOR	IDENTIFIED ANTIGEN	THERAPEUTIC/PROPHYLACTIC EFFECTS	IDENTIFIED ANTIBODIES	REFERENCE
EGFR GENE	PAK-8 M13 pVIII	EGFR mimotope Triple tandem repeat	Decreased tumor growth Humoral response Lewis lung carcinoma tumor model High level of cytokines Anti-cancer activity	Anti EGFR monoclonal antibody ICR62	[28,28]
*MYCOBACTERIUM LEPRAE*	M13 (pIII)	Anti *M.leprae* epitopes	High antibody titer High immunogenicity in mice	Human antiserum	[83,83]
IXODES SCAPULARIS TICKS SALIVARY GLAND	pHORF3 M13	Metalloprotease (MP1)	No evaluated	3 biopanning rounds with human serum antibodies against salivary gland homogenate (SGH)	[84,84]
*LEISHMANIA INFANTUM* *(SYN. L. CHAGASI)*	M13	Peptide 5	Protective effect vs. *L. infantum* in mice model High immunogenicity	Polyclonal IgGs from *L. infantum* infected dogs	[85,85]
SALMONELLA TYPHIMURIUM	pHORF3 M13	Novel immunogenic antigens	High immunogenicity	Serum from infected pigs	[86,86]
*TRICHINELLA SPIRALLIS*	M13 (pIII)	Peptide 8F7	High levels of IgG1 Reduction of larvae in vaccinated mice	mAb 8F12	[19,19]

## 6 Disadvantages of using the phage display technique

Phages cannot proliferate and grow in eukaryotic hosts because in prokaryotic hosts, post-translational modifications such as glycosylation of proteins and formation of disulfide bonds are not performed, and because folding is directly related to the function of a protein, the desired function of a complex protein such as complex antibodies or some active epitopes on the phage surface is not possible. Also, although phage particles are potentially harmless to humans and animals, the oral route of administration can infect intestinal bacteria and cause dysbiosis, as well as release endotoxins from infectious bacteria, increasing the risk of damage to the host. This issue can be solved using non-lytic phages [76,87,88].

## 7 Bacteriophage-delivered DNA vaccines

**Fig. 7:**
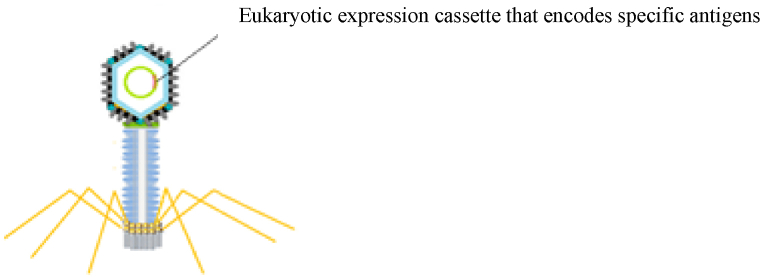
Schematic illustration of phage DNA vaccines.

The limitations of DNA vaccines, including low immunogenicity and the need for adjuvants, were solved through the application of delivery vectors and bacteriophage-DNA vaccines. Phage particles are strong and appropriate adjuvants, so they are regarded as a good vehicle for delivering DNA. The bacteriophage-DNA vaccine comprises a eukaryotic expression cassette that encodes specific antigens and is under the control of target-specific promoters [76,89]. The expression cassette should contain regulatory elements for proper antigen expression and folding (Fig. 7) [6]. The most commonly used phages for this purpose are lambda phages, but filamentous phages have also been used [90,91]. One of the significant features of filamentous phage-DNA vaccines is that the vectors can carry several gene copies simultaneously, which leads to the stimulation of immune responses against several different antigens simultaneously using only one delivery vector [90]. Bacteriophage-delivered DNA vaccines have advantages over other types of vaccines, including easy production, low cost, high safety, mass production, high stability (resistant to enzymatic digestion, high temperatures, and pH changes), the ability to carry large DNA fragments (up to 20 kb in lambda phage), and the ability to elicit an effective immune response by properly presenting antigens to immune cells [10,42,76,89,92]. Table 3 describes the bacteriophage-DNA vaccines currently developed.

**Table 3:** Description of antigen, carrier phage type, DNA cloning vector, post-administration effects, selected promoter, model of study, and dose of some antiviral phage DNA vaccines.

Vaccine	Phage type	Promoter	DNA Cloning Vector	Model of Study	Dosage	Effects after administration	Reference
Anti-viral Small surface antigen (HBsAg) of *hepatitis B*	Λ	n.r	pRcCMVHBs(S) λ-gt11	Rabbits	4 × 10^10^ pfu	Strong antibody response (IgG, IgM)	[89,89]
Anti-viral Human papillomavirus (HPV)-16 E7	λ ZAP	CMV	n.r	C57BL**/**6 mice	2 × 10^12^particles	Decreased tumor size	[41,41]
Anti-viral *Hepatitis B* Surface antigen (HB)	Λ	CMV	λ-gt11	Mice and rabbits	Mice: 5 × 10^9^ pfu Rabbits: 4 × 10^10^ HBsAg pfu	Anti HB response	[92,92]
Anti-viral *Herpes simplex* *virus 1 (HSV-1*) glycoprotein D	M13	Human cytomegalovirus immediate-early	pcDNA3-gD plasmid	Mice	1.4 × 10^15^ pfu	Anti-HSV-1 neutralizing antibodies and CytotoxicT cell response	[90,90]

pfu: Plaque forming units, CMV: Cytomegalovirus, n.r.: not reported.

## 8 Hybrid bacteriophage vaccines

**Fig. 8:**
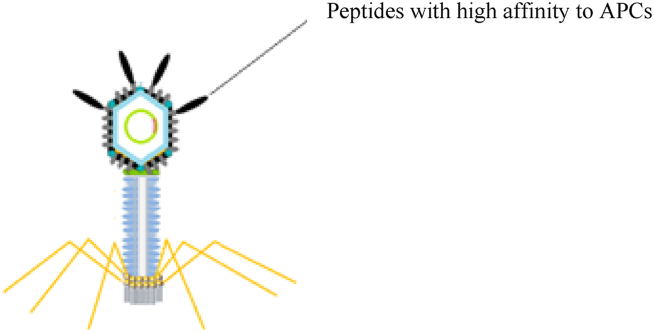
Schematic illustration of Hybrid phage vaccines.

Hybrid phage-DNA vaccines are a combination of phage-display vaccines and bacteriophage-delivered DNA vaccines. High-affinity peptides to Antigen-Presenting Cells (APCs) or the antigen itself are displayed on the phage surface, and at the same time, they harbor an eukaryotic expression cassette that contains the DNA sequences encoding specific antigens (Fig. 8) [6]. The feature enhances effective humoral and cellular immune responses [76]. In 2008, a double-hybrid filamentous phage (fd) was developed whose co-displayed peptides were recognized by the Major Histocompatibility Complex (MHC) class I and class II cell surface receptors and epitopes from MAGE antigen with the aim of enhancing T cell-based antitumor activity. Therefore, hybrid phage-DNA vaccines were proposed as a good choice for more effective anticancer vaccines [67].

## 9 Immunity against a phage-based vaccine

**Fig. 9:**
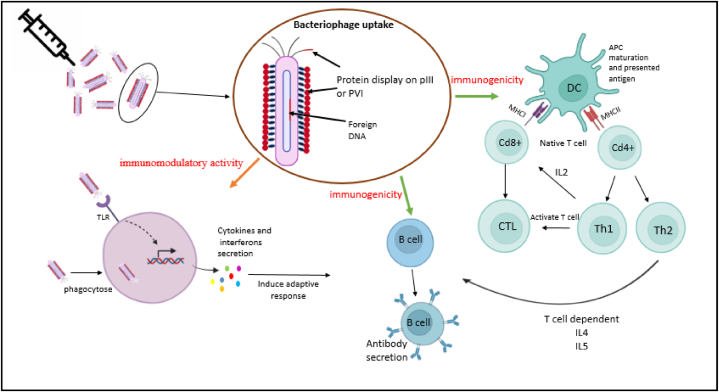
A schematic picture of how phage peptide vaccines stimulate immunity. Phages that are used in phage vaccines are engineered to display peptides designed to stimulate the immune system. These phage vaccines activate innate immune responses by macrophages, granulocytes, and complement proteins. Phage components including CpG motifs on DNA and LPS stimulate several PRRs, such as TLRs 3, 4, 7, 8, 9, and 13, on the other hand, cytokines like IL6 - IL12, etc. are produced by phagocytes to induce adaptive immune responses. Displayed recombinant protein is presented by APCs such as dendritic cells (DCs) via Major Histocompatibility Complex Class II (MHC II) to T helper cells (Th), causing to activate memory B and T cells.

The most crucial aim of vaccines is to elicit the immune system's response against pathogens such as bacteria, viruses, parasites, and fungi; therefore, it is vital to find the best vaccine that efficiently activates both immune systems' arms. After bacteriophage discovery, the interaction between phages and the immune system of mammalian cells was precisely studied [[93], [94], [95]]. The crosstalk between the immune system and phage can be studied from two distinct perspectives: phage immunomodulatory activity and phage immunogenicity (Fig. 9). Immunomodulatory phage activity is a nonspecific effect of phages to induce an innate immune response and also help to increase specific immune responses via the natural ability of phages to elicit the adaptive immune system [93,96].

### 9.1 The phage's immunomodulatory activity

#### 9.1.1 Innate immune system and cytokine secretion

Innate immunity is the body's first line of defense against pathogens. Intrinsic immunity includes phagocytes (macrophages and dendritic cells), granulocytes (eosinophils, basophils, neutrophils, mast cells, and NK cells), and complement proteins, which contribute to phagocytosis [93,97,98]. Mammalian cells express receptors called pattern recognition receptors (PRPs), which either bind to specific signals such as pathogen-associated molecular patterns (PAMPs) or identify damage-associated molecular patterns (DAMPs), such as specific motifs on the genome of foreign agents or proteins released from pathogens. One of the principal PRPs is toll-like receptors (TLRs), of which, TLR3, 7, 8, 9, 13 identify the viral genome and activate the innate immune system. Bacteriophages trigger immune responses via extracellular, endosomal, or cytoplasmic PRPs. Following the phage 's entrance via transcytosis, the phage is exposed to macrophages, dendritic cells, and mast cells. Moreover, the interaction of phages with immune cells is possible due to the circulation of phages in the blood after their entrance into the spleen. The unmethylated deoxycytidylate phosphate deoxyguanylate (CpG) sites in the phage genome act as PAMPs detected by TLR9 and activate downstream signaling pathways leading to the secretion of inflammatory cytokines. The inflammatory cytokines recruit the immune cells to the infection site and thus enhance the immune responses [93,94,96,[99], [100], [101]].

### 9.2 The phage 's immunogenicity

#### 9.2.1 Humoral immunity

Phages express immunogenic peptides or mimotopes on their surfaces that lead to direct or indirect activation of B cells by Th2 cells. Several studies have shown that the injection of M13 phage into the mouse models induced an efficient IgG antibody response even in the absence of excipients [96,102]. Researchers have analyzed the antibody responses induced by phage M13 in mouse models through MyD88 deletion to investigate the adaptive immune response. The authors have studied the initial IgG responses induced by the M13 phage in mice on day 13 after phage administration. They reported that, in contrast to wild-type mice, the mice carrying the MyD88 deletion did not respond to IgG antibodies. Results showed that phage M13 triggered an efficient antibody response consisting of IgG2b, 2c, and 3, through MyD88 [93,103].

#### 9.2.2 Cell immunity

T cells, including both CD8^+^ and CD4^+^ T cell subsets, play essential roles in viral infections and in preventing the growth and metastasis of tumor cells. The activation of CD8 + T cells and engagement of MHCI are of great importance to fighting against viral infections and one of the goals of vaccine design. Although the efficacy of phages on the activation of cellular immunity is based on a broader range of studies, it has been shown that the epitopes of phages can activate cellular immunity as well. For instance, the expression of the rt2 peptide, derived from the HIV reverse transcriptase enzyme on phage f2, activates cytotoxic T lymphocytes (CTLs) in human cells. As mentioned above, the phage 's entrance as a foreign particle triggers the antigen-presenting cell (APS) to process and present the epitopes to T cells [101,[103], [104], [105]].

Filamentous bacteriophages displaying peptide epitopes can be administered by oral or parenteral means. Although the parenteral administration exhibits robust immunogenic features the same as the oral or intranasal administration, the amount of serum IgA and IgG was higher in the oral and parenteral administrations when compared to the intranasal administration [106,107]. In another aerosol-based strategy, a short sequence of the pIII protein was used to target and transport the phage particles into the systemic circulation. The results revealed that the intranasal route of immunization might exhibit biologically significant advantages. Moreover, inhalation is needle-free and non-invasive, eliminating the requirement for specialized medical staff. Additionally, rapid access to the upper and lower respiratory tracts reduces viral shedding and subsequently lowers transmission [108].

## 10 Application of the phage vaccine

### 10.1 Anti-parasite phage-based vaccines

Various studies evaluated the application of phage-based vaccines against parasitic diseases. Bacteriophages were first applied to stimulate the immune response against parasite peptides in 1988. In this study, repeated sequences of the circumsporozoite (CS) gene of the human malaria parasite, Plasmodium falciparum, were inserted into the pIII gene of the filamentous bacteriophage, f1. Immune responses against the CS protein were reported upon administration of the engineered phage in mice and rabbit models (Table 4) [109]. As a cysticercosis vaccination, three *Taenia solium* antigens were produced on pVIII phage M13 (S3Pvac). A randomized trial on a pig population was used to compare the effectiveness of S3Pvac-Phage with a placebo. Tongue examination and necropsy results from three to five months after oral administration of the vaccine revealed that vaccination resulted in a 70% decrease in the frequency of tongue cysticercosis, a 54% decrease in muscle cysticercosis, and an 87% decrease in the total number of cysticercoses (Table 4) [15]. A 28 kDa glutathione S-transferase (Sm28GST) antigen from the human parasite *Schistosoma mansoni* was produced by Kakuturu V. N. Rao et al., in 2003. IgG2b, IgG3, and IgM antibody levels were shown to be considerably greater in the sera of inoculated animals compared to pre-immune sera in subsequent immunization trials. The levels of IgG1 and IgG2a, however, did not significantly differ from pre-immune values. Anti-M13 antibodies were used to measure the levels of anti-phage antibodies in the serum samples, and results showed that these antibodies were present in all inoculated animals two weeks following immunization. Six weeks after vaccination, 100 *S. mansoni* cercariae were given to each mouse as a challenge. Only around 30% of the population was protected after a single challenge. This may be due to the fact that Sm28GST is not the only antigen present in irradiation cercariae that contributes to the development of protective immune responses [110]. Another study tested the capacity of *fd* phages against infection with the human protozoan *Trypanosoma cruzi*, the etiologic agent of Chagas Disease. In this study, the OVA257–264 peptide or the *T. cruzi* immunodominant peptides, PA8 and TSKB20, were inserted into the *fd* phage. The results demonstrated fd phages as a potent delivery system, activating both humoral and cellular-mediated responses against *T. cruzi* infection through a TLR9-dependent mechanism (Table 4) [111].

**Table 4:** Application of phage-based vaccines.

Vaccine application	Model of study	Main Effects of Phage-Based Vaccine Administration and Comments	Phage used	Protein of pathogen that displayed	Protein Used for Phage Display	reference
Parasites						
*Plasmodium falciparum*	mice and rabbits	Elicitation of responses to the fusion protein	f1 phage	Repeat regions of the circumsporozoite (CS) protein gene	pIII	[109]
*Taenia solium*	Pig	Induced antigen-specific cellular immune responses in pigs	M13 phage	Three peptides (KETc1, KETc12, GK1) and a recombinant antigen KETc7	pVIII	[63]
*Trypanosoma cruzi*	C57BL/6 (B6) and Tlr9−/− mice	Induces anti-PA8 and antiTSKB20 IgG production, expansion of Ag-specifc IFN-γ, TNF-α, and Granzyme B-producing CD8^+^ T cells, as well as in vivo Ag-specifc cytotoxic responses	f1 phage	OVA257–264 peptide or the T. cruzi-immunodominant peptides PA8 and TSKB20	pVIII	[111]
**Viruses**						
*hepatitis B*	BALB:c (H-2d) mice	Inducing MHC class I restricted cytotoxic T lymphocytes response in vivo	Filamentous phage	Epitope S28–39	pVIII	[112]
*SARS-COV-2*	Swiss Webster or BALB/c	Enhanced a systemic and specific spike (S) protein-specific antibody response	Filamentous phage	Epitopes of the SARS-CoV-2 spike (S) protein and signal prptide	pVIII and pIII	[108]
*FMDV*	BALB/C mice	Induce high levels of IFN-γ levels in mice 31 with little effect on IL-4 levels and produce high levels of anti- 33 FMD antibodies	T7 phage	The capsid protein VP1 of the 22 OHM-02 strain, and the recombinant VP1 phage was termed OHM-T7		[113]
*HIV*	6–8 week-female BALB/c mice	The displayed p24 was highly immunogenic in mice in the absence of any external adjuvant, eliciting strong p24-specific antibodies, as well as Th1 and Th2 cellular responses with a bias toward the Th2 response	T4 phage	HIV antigens, p24-gag, Nef, and an engineered gp41 C-peptide trimer	Hoc-capsid	[114]
*porcine Circovirus 2 (PCV2)*	pigs	The LDP-D-CAP elicited both cellular and humoral immune responses	Phage lambda	Capsid protein (LDP-D-CAP)	Carboxyl-terminal of lambda head protein D	[115]
*herpes simplex virus 1(HSV-1)*	6–8 week-female BALB/c mice	In both arms of immune responses induced by recombinant filamentous phage inoculation	Recombinant phage	Glycoprotein D		[116]
*Respiratory Syncytial Virus*	5–6 week old BALB/C mice	Inducing a high level of circulating RSV-specific antibodies	Fd phage	Epitope 173–187 from the glycoprotein G	pIII	[117]
**Bacteria**						
*Bacillus anthracis*	rabbit	Activated strong anthrax- and plague-specific immune responses specially T-helper 1 and 2	T4 phage	anthrax protective antigen (PA) (83 kDa)	The small outer capsid protein Soc (9 kDa)	[118]
*Yersinia pestis*	rabbit	Activated strong anthrax- and plague-specific immune responses specially T-helper 1 and 2	T4 phage	The mutated (mut) capsular antigen F1 and the low-calcium-response V antigen of the type 3 secretion system	The small outer capsid protein Soc (9 kDa)	[118]
**Fungies**						
*Sporothrix globosa*	6- to 10-week-old BALB/c mice	Induce Gp70-specific antibody production in mice, which can in turn bind with Gp70 and treat the infection	Phage vector fuse-55	Epitope peptide (kpvqhalltplgldr) of Gp70	pIII	[119]
*Candida albicans*	6–8 week-old female BALB/c mice	Produce strong immune response as rSap2 and generate antibodies that can bind Sap2 and CA to inhibit the CA infection and increases the survival rate of CA-infected mice	Filamentous phage	aspartyl proteinases 2 (Sap2)	pVIII	[120]
**Other application**						
Contraceptive vaccine	Male BALB/c mice	Specific anti-GnRH antibody faster than conventional vaccine Spermatogenesis suppression	T7 phage	Gonadotrophin Releasing Hormone (GnRH) fragment 43 kDa	10 B	[121]
cocaine addiction vaccine	Rat		Filamentous phage	Cocaine-binding proteins (antibody-displaying)	pVIII	[122]

A potential phage-based vaccine candidate's immune response against *R. microplus* (the cattle tick) was assessed. The *R. microplus* Bm86 protein epitopes, Sbm7462, and a truncated pIII protein were displayed on the M13 phage to develop the vaccine. In an ex vivo experiment, the Sbm7462 phage vaccination induced the maturation of bovine monocyte-derived dendritic cells. Peripheral blood mononuclear cells (PBMC) from the spleen proliferated and produced antibodies against the Bm86 and Sbm7462 antigens in subcutaneously vaccinated mice. So according to the findings of the study on cattle tick antigen, phage-based vaccines induced antibody production in such a way that was higher on day 25 than on day 12 after injection [123].

### 10.2 Anti-viral phage-based vaccines

There are many diseases in animals and humans of viral origin, and phage vaccines have also been successfully developed against them. *Hepatitis B* was the target of the first phage vaccination against viruses. In order to develop a Hepatitis B hybrid phage vaccine, the *hepatitis B virus* S28-39 epitope was cloned into the pC89 phagemid vector in frame with the major envelope protein (pVIII) of M13. Studies on the immunization of mice revealed that the injection can cause certain cytotoxic T lymphocyte (CTL) responses (Table 4) [66]. Additionally, the hepatitis B core antigen gene (HBcAg) was inserted into the minor envelope protein (pIII) gene of M13. Following vaccination, ELISA results revealed that recombinant phages and recombinant phages prepared in incomplete Freund's adjuvant (IFA) were both effective vaccines that caused powerful immunological reactions. Phages alone or in combination with IFA did not significantly alter the immune response, demonstrating that the phages themselves are antigenic and trigger an immunological response [124].

Soc and Hoc have been employed to express antigens on the surface of T4 since they are not required for phage infectivity [125]. Via Hoc-capsid interactions, *HIV* antigens, p24-gag, Nef, and a modified C-peptide trimer of gp41 are presented on the T4 phage capsid surface. The human immunodeficiency virus *(HIV)* genes were attached to the 5′ or 3′ end of the Hoc gene to achieve in-frame integration. All of the accessible capsid binding sites were effectively saturated by the efficient presentation of single or several antigens. In the absence of any external adjuvant, p24 exhibited in mice was very immunogenic and produced potent p24-specific antibodies as well as Th1 and Th2 cell responses, with a preference for Th2 responses (Table 4) [114]. Li and colleagues discovered that influenza virus 3M2e proteins exhibited on T4 nanoparticles create extraordinarily high levels of 3M2e-specific IgG antibodies in the absence of any adjuvant, while 3M2e coupled to RB69 Soc only causes low amounts of 3M2e-specific IgG antibodies [126]. Among other viruses that have been used for this purpose are *Herpes simplex virus 1 (HSV-1)* (Table 4) [116], *Circovirus 2 (PCV2)* (Table 4) [115], *FMDV* (Table 4) [113], *Zika virus*, and *RSV* (Table 4) [117], which have been displayed on various phages and elicited a strong antibody response in mice when compared to non-immunized mice. Recent studies have also used phage vaccines to prevent coronavirus species, for instance, by displaying the S protein's epitope 4 of *SARS-CoV2* on protein VIII of the Filamentous phage display vector f88-4, while the capsid protein pIII included the peptide CAKSMGDIVC. The transport peptide CAKSMGDIVC can identify lung epithelial cells in the lung. Recombinant phages were successfully applied to activate an antigen-specific humoral response against *SARS-CoV 2*, thus representing an appropriate candidate for vaccine development against COVID-19 (Table 4) [108]. Furthermore, vaccines using antigens other than the S protein from *SARS-CoV-2* have been developed. While the nucleocapsid protein (NP) was inside the phage, the ectodomain trimers of the S protein were produced on the T4 phage to develop T4-CoV-2. A *SARS-CoV-2* vaccine administered intravenously boosted the immune response on mucosal surfaces in comparison. The intramuscular injection, however, did not show this. Moreover, this was noticed in transgenic mice for the human angiotensin-converting enzyme (hACE2). These mice were resistant to the original *SARS-CoV-2* and its delta form up to a lethal dose. The vaccine did not harm the microbiome and was stable at room temperature [61]. Zhu et al. created a platform to develop various *SARS-CoV-2* vaccine candidates utilizing CRISPR editing of the T4 phage. Several phage segments were constructed using various SARS-CoV-2 antigens (such as the S, E, and NP proteins). Mice were shielded from *SARS-CoV-2* infection by T4 adorned with S-trimers, which generate particular antibodies and prevent the binding of RBD to ACE-2 [127].

The safety of ABNCoV2, a vaccine based on VLPs of bacteriophage AP205 adorned with RBD of *SARS-CoV-2* generated in S2 Drosophila cells, was assessed in healthy volunteers during a clinical phase I/II experiment (NCT04839146) [128]. Clinical trials.gov has not yet published the study's results, although it ended on February 25, 2022. ABNCoV2 is also the subject of an open-label phase 2 trial in Germany (NCT05077267; EUCTR2021-001393-31). Adults who had previously received the *SARS-CoV-2* vaccine underwent a phase three trial (NCT05329220) to assess the immunogenicity, safety, and tolerability of ABNCoV2 [128].

### 10.3 Anti-bacterial phage-based vaccines

One of the most critical issues in bacterial infections is antibiotic resistance due to excessive use of antibiotics. The phage-based vaccine is introduced as a reliable approach to overcoming bacterial antibiotic resistance. The phage-based vaccines have been developed against *Bacillus anthracis* and *Yersinia pestis's* antigens, the causative agents of anthrax and plaque, respectively. This phage-based vaccine elicited strong anti-anthrax and plague-specific immune responses, especially T-helper 1 and 2 cellular immune responses, which are essential for eradicating bacterial infection (Table 4). Administration of phage-based vaccines in animal models (mice, rats, and rabbits) infected with lethal doses of both anthrax lethal toxin and *Y. pestis* CO92 bacteria revealed complete protection. Therefore, phages like T4 could be applied with nanoparticles to formulate multivalent vaccines against high-risk pathogens and emerging infections [109,118].

*Chlamydia trachomatis*, a bacterium found in the mouth, genital tract, cervix, and urethra of adults, was studied as a candidate for a phage vaccine. The Q-CT584 vaccine was developed by chemically attaching epitopes 70–77 and 154–164 of CT584 to bacteriophage Q VLPs. CT584, a protein that binds to the type three secretion system (T3SS) involved in invasion, intracellular survival, and cell egress, was selected. Mice immunized intramuscularly with Q-CT584 and then challenged with *C. trachomatis* produced large amounts of IgG that prevented infection [129]. Another example of a phage vaccine against bacteria is cholera disease. Cholera is an acute, secretory diarrheal disease caused by the highly pathogenic bacterium *Vibrio cholerae*. Only the serogroups O1 and O139 may result in epidemic cholera out of the more than 200 serogroups. By chemically joining the antigen O-specific polysaccharide (OSP) isolated from *V. cholerae* O1 El Tor Inaba to VLP produced from phage Q, a vaccine (Q-OSP) against *V. cholera* was developed. When mice were immunized with the Q-OSP formula, high and enduring levels of IgG antibodies were produced against OSP. These antibodies recognized the natural LPS from *V. cholerae* O1 El Tor Inaba. Moreover, the live bacteria could be killed by antibodies in sera from mice that had received complement vaccinations [130].

### 10.4 Anti-fungal phage-based vaccines

One of the applications of phage is the induction of an immune response against fungal infections. Although there is still no approved antifungal phage-based vaccine for humans, there have been several successful studies regarding anti-fungal bacteriophage vaccines. *Candida albicans* (Table 4) [120] and *Sporothrix globose* (Table 4) [119] are the fungal agents that the phage vaccines have developed against. Immunization with recombinant phage increased the survival rate of mice following each fungal infection, separately.

### 10.5 Anti-cancer phage-based vaccines

Cancer vaccines, regarded as promising tools for cancer treatment, have recently received increased attention. The expression of antigens fused to phage surface proteins in the phage display system is intended to induce tumor-specific T-cell responses [93]. Cross_presentation is a strategy by which phages can induce antigen presentation mediated by MHC-I and MHC-II molecules. The cross-presentation is also extremely useful in developing cancer immunotherapies, as cytotoxic T lymphocyte (CTL) activation by MHC-I recognition is critical for tumor cell death [131,132]. In recent years, many tumor-associated antigens that CTLs recognize have been identified and characterized. Cancer/testis (C/T) antigens are the fastest-growing group of tumor-associated antigens, expressed in various types of tumors but not in normal tissues [133,134]. As a result of their tumor-specificity, C/T antigens are known as appropriate candidates for cancer vaccines [135]. Sartorius and colleagues used the filamentous *fd* phage to co-express T helper (Th) cell-specific epitopes and C/T antigens to elicit Th-dependent CTL responses. The recombinant phage could elicit potent, specific CTL responses in vitro and in vivo. The filamentous phages enhance the immunogenicity of tumor-associated antigens while simultaneously slowing tumor growth [136].

In a similar way, another study used recombinant M13 phage as a vaccine for Lewis lung carcinoma. The pVIII coat protein showed an epidermal growth factor receptor (EGFR) mimotope. This study demonstrated that recombinant phage vaccines could significantly induce immune responses and elicit specific antibodies against cancer cells [18]. Non-filamentous phages such as T4 and T7 have also been used as vaccines to improve cancer therapy after exposure to appropriate peptides. For instance, T4 phage was used to express vascular endothelial growth factor (VEGF), and subsequently, the engineered age was applied as a vaccine against Lewis lung carcinoma in a mouse model. The T4-mVEGFR2 phage could effectively suppress angiogenesis and have significant anti-tumor activity. Furthermore, the T7 phage displays the five fragments of the EGFR mimotope, which were fused with the T7 phage's 10B coat protein via genetic engineering. The results showed that the EGFR was successfully expressed on the surface of the T7 phage, and thus the recombinant phage could effectively inhibit tumor growth in the BALB/c mouse model [32]. The discovery of tumor-specific antigens opens the way for the successful development of phage-displayed vaccines for cancer prevention. Shadidi et al. for example, used a proteomics-based method to identify breast cancer tumor antigens and created vaccines by displaying the identified tumor antigens on the T7 phage and measuring the immune responses in mice after oral administration. Their findings showed that the phage's surface display of tumor antigens could effectively elicit immune responses, thus introducing the engineered phage as a promising mucosal cancer vaccine [137].

n the fd phage display system, a tumor-specific antigen epitope called melanoma antigen A1161169 was joined to the pVIII coat protein of the fd phage. An in vivo tumor protection assay confirmed that the hybrid phage effectively inhibited tumor growth. These studies clearly showed the potency of phages for engineering an effective vaccine and improving cancer therapy [70]. Recently, an immunogenic bacteriophage-based vaccine with HER2/neu overexpression stimulated CTL activity. This study revealed the phage nanoparticles containing GP2 as a fused peptide to the gpD phage capsid protein, elicited a strong CTL response, and protected mice against HER2/neu-positive tumors [138].

The Δ16HER2 is a HER2 splice variant and the transforming isoform in HER2-positive breast cancer. Δ16HER2 has been shown to promote breast cancer aggressiveness and is a leading factor in drug resistance. However, due to tolerogenic mechanisms against the human HER2 self-antigen, a situation frequently observed in HER2+ patients, the vaccines failed to elicit immunological protection in Δ16HER2 transgenic mice. An engineered bacteriophage with immunogenic Δ16HER2 epitopes was used as an anticancer vaccine in a recent study. Through the disruption of immunological tolerance, these phage-based vaccinations were able to elicit a protective anti- Δ16HER2 humoral response. Altogether, these findings support the practice of phage-based anti-HER2/Δ16HER2 vaccination as a safe and effective immunotherapy strategy for HER2-positive breast cancers [139].

Unexpectedly, many promising preclinical investigations that could have led to clinical trials have not been repeated, and we are unsure of the reason for this. Hence, more work is needed to advance this technology, which has a lot of benefits and great promise, to the next stage of clinical trials, where it can be shown effective for usage in both humans and animals. The use of phage-based vaccinations is advantageous because several clinical trials have demonstrated that phages are both safe for humans and animals. The encouraging findings of Roehnisch in 2014 serve as a solid springboard for expanding the use of phage-based vaccines in clinical trials, at least for those based on M13 that have demonstrated safety [140]. This viewpoint is positive since it suggests that phages may eventually be used in vaccinations, which is possible based on the information presented above [62].

### 10.6 Other types of diseases or conditions

Another application of phage-based vaccines is in population control using the immune contraceptive vaccine, which induces an adaptive immune response against the reproductive system and causes temporary infertility. For instance, the gonadotrophin-releasing hormone (GnRH) antigen was displayed on the surface of T7 phages in a mouse model leading to immune castration [121]. Vaccine targeting could also be applied to drug addiction by using bacteriophages that display antibodies that block the effects of various drugs, such as cocaine [122].

A group of neurodegenerative illnesses known as tauopathies includes frontotemporal dementia (FTD) and Alzheimer's disease (AD). Tauopathies are brought on by neurofibrillary tangles (NFTs), which are formed in neurons by hyperphosphorylated pathogenic tau (pTau). Tau may therefore be a useful target for vaccination to prevent these illnesses. The development of pT181-Q, a phage-based vaccine against tauopathies, involved conjugating a tau peptide to bacteriophage Q's VLPs. Animals given the pT181-Q vaccine produced a strong IgG reaction against pT181. In mice immunized with pT181-Q, less pTau accumulated in the brain and hippocampus. The amount of circulating CD3^+^ T-cells and neuroinflammation in the brain both decreased [141].

Another application of phage vaccines is in heart disease. A higher risk of cardiovascular disease is linked to low-density lipoprotein cholesterol, which contains three checkpoint proteins (PCSK9, ApoB, and CETP). The three proteins were displayed on the bacteriophage Q coat protein VLPs to develop a phage-based vaccination. Immunized mice produced IgG1 and IgG2b immunoglobulin isotypes against PCSK9, ApoB, and CETP. The immunizations also lower the levels of these proteins, which lowers the plasma's overall cholesterol level [142].

## 11 Conclusion and future perspective

Phage vaccines may provide the key to developing innovative methods for battling cancer and other bacterial and viral infections. The global abundance of phages and our increased understanding of how to exploit them will undoubtedly lead to the emergence of new phage display systems. This overview discusses the most recent developments in the field of phage-based vaccines and their capacity to be used as a preventative and therapeutic platform for a variety of diseases. It also covers the potential directions for advancing this field. Although there have been some breakthroughs with phage-based vaccinations, limitations still need to be resolved to improve this field. Without a doubt, phage display cannot overcome all hurdles that stand in the way of the development of vaccines and is unable to address all issues that may arise in the way of vaccine design and production. Different phage-based vaccine platforms have a promising future, and it can be claimed that phage display's importance will only increase over the next few years.

## Declaration of competing interest

The authors declare that they have no known competing financial interests or personal relationships that could have appeared to influence the work reported in this paper.
